# Feasibility and Conceptualization of an e-Mental Health Treatment for Depression in Older Adults: Mixed-Methods Study

**DOI:** 10.2196/10973

**Published:** 2018-10-23

**Authors:** Christiane Eichenberg, Markus Schott, Adam Sawyer, Georg Aumayr, Manuela Plößnig

**Affiliations:** 1 Sigmund Freud Privat Universität Wien Vienna Austria; 2 Johanniter Österreich Ausbildung und Forschung gemeinnützige GmbH Vienna Austria; 3 Salzburg Research Forschungsgesellschaft mbH Vienna Austria

**Keywords:** depression, online therapy, e-mental health

## Abstract

**Background:**

Depression is one of the most common mental disorders in older adults. Unfortunately, it often goes unrecognized in the older population.

**Objective:**

The aim of this study was to identify how Web-based apps can recognize and help treat depression in older adults.

**Methods:**

Focus groups were conducted with mental health care experts. A Web-based survey of 56 older adults suffering from depression was conducted. Qualitative interviews were conducted with 2 individuals.

**Results:**

Results of the focus groups highlighted that there is a need for a collaborative care platform for depression in old age. Findings from the Web-based study showed that younger participants (aged 50 to 64 years) used electronic media more often than older participants (aged 65 years and older). The interviews pointed in a comparable direction.

**Conclusions:**

Overall, an e-mental (electronic mental) health treatment for depression in older adults would be well accepted. Web-based care platforms should be developed, evaluated, and in case of evidence for their effectiveness, integrated into the everyday clinic.

## Introduction

In 2014, about 18.5% of the approximately 506.8 million inhabitants of the European Union were aged over 65 years. By 2050, this number is expected to rise to 28.1% [1]. The population growth in this age group is not only evident for Europe but can be observed worldwide. Depression is the second-most common type of mental illness in older people [2].

Current prevalence rates for depression range from 1% to 6% of the total population. [3] Prevalence rates (between 6% and 16%) are significantly higher among older people [4] and dementia patients [3]. The occurrence of subclinical symptoms in the elderly is much more common in comparison with clinical depression, and prevalence rates range from 10% to 16.5% [5].

Depression in old age is diagnosed according to the Diagnostic and Statistical Manual of Mental Disorders (DSM) [6] and the International Classification of Diseases (ICD) system [7]. Depressive episodes can be classified as *easy*, *moderate*, or *severe*. To meet the diagnostic criteria for depression, the depressive episode should last for at least 2 weeks. The main symptoms of a depressive episode according to ICD-10 are depressive mood, loss of interest or pleasure in activities, and a diminished drive. Additional symptoms are loss of self-confidence, unsubstantiated self-reproaches, recurring thoughts of death or suicide, difficulties in concentrating, and psychomotor agitation. Differences in clinical symptoms between elderly people with depression and younger adults with depression are very well reported. Suicide rates in the elderly are higher than in younger adults and more closely associated with depression. Depressed older adults are less likely to endorse affective symptoms and more likely to display a loss of interest than younger adults [8]. Depression in old age is associated with cognitive impairments, as well as other somatic comorbidities [9]. Many studies identified personality traits [10] and a negative self-image [11] to be risk factors for depressive symptoms and disorders. Several studies show a link between depression and quantitative and qualitative aspects of social network [12].

Only about 25% of patients with depression in old age receive psychotherapy [13]. Dakin and Areán [13] reason that because of the fact that treatment guidelines for age-related depression are often lacking, psychotherapy is seldom prescribed. Espinoza and Kaufman [14] point to further reasons why an elderly patient with depression seldom receives psychotherapy, namely, a potentially difficult access to qualified staff, limitations in mobility for older people, and the inability of therapists to tailor a therapy adequately to the elderly. The lack of psychotherapeutic care for depression in old age is being criticized, and the call for innovative treatment options is becoming bigger [15]. Early detection and treatment help to reduce symptoms, improve the quality of life, and prevent an unfavorable prognosis. Therefore, early detection of people who are likely to develop a depressive disorder is desirable. A possible solution for early detection, management, and treatment of depression in old age is an electronic mental (e-mental) health program [16]. The area is constantly evolving and has significantly expanded conventional services such as *face-to-face* interventions. E-mental health offers range from providing health-related information via Web 2.0 interventions to mobile phone apps [17]. According to Wicks and colleagues [18], e-mental health offers the following 3 particular advantages: (1) improving the efficiency of care through better communication and diagnostics possibilities while reducing costs, (2) improving quality of care, and (3) promoting active cooperation and autonomy of the patient.

A large number of innovative interventions in the e-mental health sector for the prevention and treatment of depression have been developed. E-mental health research projects are mostly focused on the monitoring of symptoms [19], the early detection of depression [20], and sensors and devices that record vital data.

Most e-mental health projects that include some form of intervention are based on cognitive behavioral therapy (CBT); therefore, they are also called computerized cognitive behavioral therapy (cCBT). The effectiveness of cCBT therapy is well documented for mental illness [21]. There are more than 100 studies on cCBT for various mental disorders [22]. Particularly, cCBT for depression and anxiety disorders is well researched and has been proven to be effective [23]. A review of cCBT in depression reported an average effect size (*d*) of 0.56 for 19 included randomized controlled trials [24]. Similarly, a study by Spek and colleagues [23] that specifically looked at elderly (aged 50 years and older) showed that cCBT was at least as effective as a commonly used group CBT intervention for subthreshold depression. In addition to the efficacy studies of individual cCBT interventions, there are now several international meta-analyses that provide sufficient evidence that computer-assisted cognitive behavioral interventions are generally effective [25]. In 17 of the 19 analyzed studies, positive evidence of effectiveness was found. Averaged across all disorder patterns and forms of intervention, the average treatment effect was 0.53, which is quite similar to effect sizes for traditional outpatient treatment [25].

Examples of cCBT include Help4Mood, an agent-based decision support system, for severely depressed patients [26]; THIS WAY UP, an Australian online training program [27]; or MasterMind, a European project that consists of a 10-week training program based on cCBT [28]. SeniorEngage is a project from the organization Second Ambient Assisted Living-Call, which has developed Web 2.0 tools for social networking to prevent isolation and depression of people in retirement [29]. Beating the Blues is an example of an English online platform with CBT as its methodical base. Beating the Blues integrates storytelling elements into its 8 modules [30]. Considering German-speaking countries, a very prominent example is the online program Deprexis, which consists of behavioral modules for behavioral activation or cognitive restructuring [31].

Unfortunately, these projects fundamentally neglect discussed age-related issues of depression. eHealth programs to reduce depressive symptoms in elderly people are not readily available despite the many benefits of such interventions. Therefore, there is a need for a collaborative care platform for people with depression in old age addressing the special circumstances and needs of the elderly, as well as the needs and requirements of those caring for them and treating them.

Therefore, the goal of this study was to identify the development of a collaborative Web-based care platform for people with depression in old age, addressing the special circumstances and needs of the elderly, as well as the needs and requirements of those caring for them and treating them. In particular, the following aspects will be examined to elucidate the research question:

1. How common and regular is the use of information and communication technology (ICT) in the elderly in general and in elderly patients with depression?
2. What are the contents and requirements for a collaborative Web-based care platform for self-management and early detection and interventions of depression in old age?
3. How willing are elderly patients with depression to use such a collaborative Web-based supply platform?

## Methods

### Study Design

In a series of 3 consecutive studies, the feasibility and conceptualization of an e-mental health treatment for depression in older adults were investigated. First, focus groups with mental health care experts were conducted. An online study was launched in forums and depression-related online platforms. Finally, to allow for more in-depth insights, a series of interviews was conducted. Ethical approval was obtained from the Sigmund Freud University Vienna ethics committee.

#### Focus Groups

Two focus group discussions were held in February and March 2016. The groups were recruited by personal contacts within medical care organizations in Austria. Focus groups were supposed to consist of a maximum of 8 and at least 3 people. A guideline prepared in advance served as a basis for the questions posed. Each focus group lasted for around 90 min. With the consent of participants, the 2 focus group discussions were recorded. After the discussions, the material was transcribed.

#### Online Study

Furthermore, an online study was conducted. The data collection period lasted 12 weeks. Filling out the questionnaire took about 15 to 25 min.

Data collection took place in 2 phases. Phase 1 started in March 2016. The questionnaire was posted in an online forum. As a result of this pretest, the questionnaire was revised regarding its practicability, comprehensibility, and completeness of item formulation. Phase 2 started a week later. The questionnaire was published in 14 online forums and websites focusing on depression or forums for the elderly.

#### Interviews

Participants of the online questionnaire were able to leave their contact data if they were willing to participate in a follow-up interview study. Participants from both samples (50- to 64-year-olds or 65-year-olds and older people) were selected for the interviews. They were then contacted and invited to participate in the interviews.

The interviews were conducted by telephone in June 2017. A guideline prepared in advance served as a basis for the questions posed in the interviews. With the consent of the participants, interviews were recorded. The recorded material was transcribed. Interviews lasted between 60 and 90 min.

### Instruments

#### Focus Groups

To secure a structured and standardized structure for the focus groups, a guideline was predefined. Information included in the guideline was the objectives of the focus groups, open questions, and explanatory remarks. Organizational information was also included. The method of qualitative content analysis according to Mayring was chosen for the analysis of transcripts.

#### Online Study

Due to a lack of research in the area being investigated, no suitable standardized scales were available to use. On the basis of a comprehensive literature search, a questionnaire was constructed. The questionnaire included the following 6 subjects:

1. Sociodemographic data and mental health status
2. Previous experience with offline as well as online therapy for depression
3. Social support
4. Media behavior
5. Self-management strategies for coping with depressive symptoms
6. Possible features of an online care platform

Two standardized tests were part of the questionnaire to assess the severity of depression and personality of participants.

The severity of the depression was assessed with the Patient Health Questionnaire (PHQ). The PHQ-9 is a 9-question instrument that takes less than 3 min to complete. Results of the PHQ-9 may be used to make a depression diagnosis according to DSM-4 criteria. As part of the PHQ-9, the severity of depression is scaled (from 0-27) and categorized (minimal, mild, moderate, and heavy). In general, a total of 10 or above is suggestive of the presence of depression. A Cronbach alpha of .89 indicates acceptable reliability [32]. Personality dimensions of participants were recorded using the 10-item Big Five Inventory (BFI-10). The BFI-10 consists of 10 items, 2 for each dimension of personality. Interviewees indicate their response on a 5-point Likert scale ranging from *does not apply at all* (rated as 1) to *fully applies* (rated as 5). It takes about 2 min to complete the BFI-10. The test is interpreted by averaging the answers on the 2 items per personality dimension. Obtained values ranged from 1 to 10. Results from multiple samples and for 2 languages, namely English and German, suggest that the BFI-10 possesses acceptable psychometric properties [33]. Statistical analyses were conducted using SPSS 23.

#### Interviews

The guideline for the half-structured interviews was developed with reference to relevant research questions that remained unanswered in the previous online survey. The following research questions were formulated:

1. What do participants know about and what are their experiences with electronic support and support?
2. Which features are essential for an online care platform to be actively used by elderly patients with depression?

For this study, the method of qualitative content analysis according to Mayring was chosen for the analysis of transcripts.

### Sample

#### Focus Group

For the 2 focus groups, 8 experts in mobile and stationary health care, mental health care, day care, and psychosocial services, as well as gerontopsychiatry were recruited (see Table 1). The first focus group consisted of 3 participants, whereas 5 experts were part of the second focus group.

#### Online Study

The final sample consisted of 56 participants, including 35 (35/56, 63%) women and 21 (21/56, 38%) men. Mean age of the participants was 61.77 years (SD 8.74). A majority of participants (43/56, 77%) came from Germany, 20% (11/56) from Switzerland, and 4% (2/56) from Austria. There were 43% (24/56) married participants, whereas 18% (10/56) were divorced, 16% (9/56) lived in a partnership, and 14% (8/56) were single. Only 5% (3/56) were widowed, and 4% (2/56) were currently living separated.

**Table 1:** Focus groups participants.

Sex	Profession
Female	Nursing director and head of palliative care
Male	Case manager at Fonds Soziales Wien
Male	Nursing director of the Vienna Social Services
Female	Head of a nursing home
Male	Psychosocial service
Male	Mobile individual nursing
Male	Mobile individual nursing
Male	Board member at the Institute of Ethics and Law in Medicine

Considering that individuals aged 55 to 64 years (66%) use the internet more than people aged 65 to 74 (45%, for people aged 74 and older there is no data available), statistical analysis was conducted separately for the 2 age groups to allow for more in-depth findings [34].

#### Interviews

Unfortunately, only 2 people agreed to participate in the interviews. A 51-year-old, divorced, early retiree participated from the age group of 50 to 64 years. The interviewee had been suffering from severe, recurring depressive episodes for more than 10 years. Person A had experience in different forms of psychotherapy. Of the sample group 65 years and older, Person B, aged 70 years participated. Person B suffered from various depressive symptoms for more than 24 years. Therapeutically, Person B made use of outpatient psychotherapy, psychiatry, and medication.

## Results

### Focus Groups

The focus groups investigated what experts consider is required for a collaborative online care platform to work and how experts rate the benefits of a collaborative internet-based care platform for older and depressed individuals. Following the analysis of the transcribed focus group material, the following 8 categories were formed: perception of depression in old age in society, factors that influence depression, self-management of depression, usability of existing internet-based interventions, support of relatives, support of therapists, definition of the potential user group for a collaborative care platform, and third parties and ethical issues.

The need to develop a collaborative care platform for depression in old age was repeatedly emphasized during both focus groups. This becomes particularly important when taking into account that use of new media will become even more commonplace in the future. In nowadays’ society, depression in old age frequently goes undiagnosed. The impact of depression on general health is underestimated. Experts emphasized that there is a plethora of illnesses that facilitate the development of a depression, such as dementia or cancer. However, social factors, for example, social isolation, cannot be ignored.

A focus group participant remarked: “I believe that many relatives are going through hell.” Taking into account that relatives of old patients with depression are often left alone and seldom get help, the experts unanimously concluded that a collaborative care-platform where caregiving family members find relief would be very beneficial. Furthermore, this could ease the therapeutic process by providing vital information to medical and psychological therapists involved.

It was questioned whether the motivation of depressed elderly was high enough to use corresponding online offers. For an internet-based collaborative care-platform to be practical, focus group participants suggested several critical factors: the system must be integrated into the lives of those affected, instructions for handling the platform need to be described accurately and adequately, and the validity of the product should be empirically proven. Experts highlighted that it is important to provide a precise definition of depression in old age, the user group, and the form and scope of the platform. Ethical and legal consequences that involve the inclusion of additional persons to support the stakeholders in the internet-based platform need to be considered.

### Online Study

#### Depressive Symptoms

More than 90% (50/56) of all participants indicated to suffer from some form of depressive symptoms at some point in their lives. For 8.9% (5/56) of the participants, depressive symptoms started in the last 12 months. Another 45% (25/56) of the participants reported having depressive symptoms for 1 to 10 years. For 35.8% (20/56) of the sample, depressive symptoms began more than 11 years ago. Results of the PHQ-9 revealed a mean score of 12.36 (SD 6.33) of the total sample (a score of 10 or above indicating depression). The mean depression score of the participants aged 50 to 64 years was 13.90 (SD 6.52). In comparison, the mean depression score of the participants aged 65 years and older was 10.58 (SD 5.73). A *t* test revealed a significant difference between the 2 groups (*t*_54_=2.03, *P*=.04, *d*=0.54). Considering the descriptive results, it can be concluded that the severity of depression is more pronounced in the younger age group. Currently, 58.8% (33/56) were in treatment for depression.

#### Personality

For the total sample, mean values for the 5 personality dimensions were between 5.11 (Conscientiousness) and 6.34 (Neuroticism), suggesting normally pronounced personality traits.

#### Use of Information and Communication Technology

Although more than half (34/56, 61%) of participants indicated to use a computer on a daily basis, about half (28/56, 50%) of participants used a mobile phone daily. Tablets were least frequently used, with 52% (29/56) of participants stating that they are not using this device at all. With regard to the purpose of ICT use, sending email (51/56, 93%) and finding information online (50/56, 91%) were most common, followed by health-related purposes (34/56, 62%) and visits to internet forums (43/56, 62%). Internet games (11/56, 20%) proved to be the least used form of ICTs. Looking at the participants from the age group 50 to 64 years, it can be seen that 83% (25/30) of this group use a computer or laptop and a mobile phone (25/30, 83%) daily. By contrast, 47% of this group said they never use a tablet. All participants (30/30, 100%) of this age group indicated that they use an ICT device at least once a week. A computer or laptop was used by only 38% (10/26) and a mobile phone by 38% (10/26) of those aged 65 years and older every day. This group also reported using tablets least frequently. Almost all participants (25/26, 96%) of this age group reported that they use ICT devices less frequently than once a week.

Correlations between the use of ICT and age groups were investigated using chi-square tests. There was a significant relationship between age groups and the use of computers or laptops (*P*<.001). As expected, participants aged 50 to 64 years tend to use computers or laptops more frequently than those aged 65 years (see Table 2). Most participants in each media category stated that their media use did not change during a depressive period. Participants who reported a change were less likely to use ICT (see Table 3). There was a significant correlation between the use of ICT for finding information and coping with depressive symptoms and the severity of depression (*r*=.346, *P*=.01); particularly, patients with a more pronounced depressive episode used ICTs more frequently.

#### Previous Experience With Offline and Online Therapy

In terms of previous experience with offline services, it can be concluded that participants had the most experience with individual psychotherapy or psychological counseling (45/56, 80%). The most popular offline support therapy option was prescription drugs (54/56, 96%). Overall, only 25% (14/56) of participants were aware of internet-based interventions. Only 4% (2/56) of the people reported that they already tried internet-based therapy. However, some participants had experience with online self-help platforms (13/56, 23%).

#### Social Support

Looking at the entire sample, friends are the main source of potential help during depressive episodes (33/56, 59%), followed by partners (25/56, 45%) and children (20/56, 36%). A total of 14% (8/56) said they did not have anyone they could reliably get their hands on. More than half (33/56, 59%) of the sample was satisfied with their current level of social support (eg, practical or emotional assistance). Another 38% (21/56) reported that the support from their friends and family was improvable, whereas 4% (2/56) wanted less social support.

**Table 2:** Use of information and communication technology.

Frequency of use		50-64 years (n=30), n (%)	65 years and older (n=26), n (%)	Total (N=56), n (%)
**Mobile phone**				
	On a daily basis	18 (60)	10 (38)	28 (50)
	More than once a week	3 (10)	3 (12)	6 (11)
	Once a week	1 (3)	0 (0)	1 (2)
	Less frequently than once a week	0 (0)	1 (4)	1 (2)
	Never	8 (27)	12 (46)	20 (36)
**Tablet**				
	On a daily basis	5 (17)	3 (12)	8 (14)
	More than once a week	7 (23)	5 (19)	12 (21)
	Once a week	1 (3)	2 (7)	3 (5)
	Less frequently than once a week	3 (10)	1 (4)	4 (7)
	Never	14 (47)	15 (58)	29 (52)
**Computer**				
	On a daily basis	25 (83)	9 (35)	34 (61)
	More than once a week	2 (7)	4 (15)	6 (11)
	Once a week	1 (3)	5 (19)	6 (11)
	Less frequently than once a week	2 (7)	2 (8)	4 (7)
	Never	0 (0)	6 (23)	6 (11)

**Table 3:** Change of media use during depressive episodes.

Media		n (%)
**Mobile phone**		
	Less likely	20 (35)
	No change	29 (51)
	More frequently	8 (15)
**Tablet**		
	Less likely	25 (44)
	No change	27 (49)
	More frequently	4 (7)
**Computer**		
	Less likely	14 (25)
	No change	30 (53)
	More frequently	12 (22)

**Figure 1:**
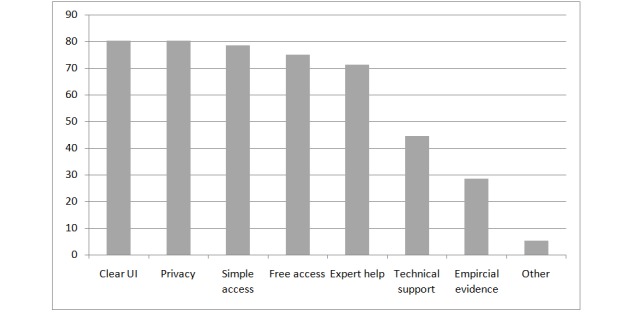
Prerequisites for a collaborative online care platform. UI: user interface.

#### Prerequisites for a Collaborative Online Care Platform

For most participants (45/56, 80%), online privacy as well as a clear user interface (45/56, 80%) were of particular importance. Other factors that were considered important included simple (44/56, 79%) and free access (43/56, 75%) to the platform. By contrast, scientific evidence for the effectiveness of the platform was important only for 29% (16/56) of the sample (see Figure 1).

For 91% (51/56) of participants, the most important content a potential platform should have was *self-help strategies* for coping with depressive symptoms. Similarly, 88% (49/56) thought that *Information on Early Detection* of depressive symptoms should be included. On the other hand, only 45% (25/56) of the participants considered *self-management applications* (eg, online diary of positive and negative thoughts) important or very important. Furthermore, 57% (32/56) of respondents indicated that *internet-based psychotherapy* (eg, online behavioral therapy to treat depression) is important or very important for a platform. A majority (47/56, 84%) of participants thought that a delineated part to which only those affected and other authorized persons (eg, therapist or general physician) would have access would be helpful. A further 41% (23/56) of participants welcomed a separate part for relatives of the patient.

#### Willingness to Use a Collaborative Online Care Platform

Willingness to use a collaborative care platform of participants was assessed with 6 items (3 positive and 3 negative poled items). Three-quarters (42/56, 75%) of the participants indicated that it was likely that they would register for and use an online care platform. When considering the 2 distinct age groups, a majority of the participants aged 50 to 64 years (27/30, 90%) said they would be willing to register and use an online care platform. Among those aged 65 years and older, 58% (15/26) indicated that they would register for and use an online care platform. Whether a platform would be helpful in managing depressive symptoms was answered positively by 80% (45/56) of the sample. Another 91% (51/56) believed that online care platforms could enable more people to connect with fellow sufferers and professionals.

A *t* test was conducted to investigate the differences in the willingness of the 2 groups to use a collaborative care platform (*t*_54_=3.59, *P*=.002, *d*=0.94). Considering that Levene test indicated unequal variances (*F*_54_=6.831, *P*=.01), findings have to be interpreted with care. It was found that 50- to 64-year-olds (mean 3.08 [SD 0.42]) were significantly more willing to use an online platform than those in the age group of 65 years and older (mean 2.56 [SD 0.66]). Interactions between willingness to use and other variables were examined. There was a positive correlation between the current use of ICT for depression-related purposes and the willingness to use an online care platform (*r*=.442, *P*<.001). Unsurprisingly, current users were more willing to try out another online platform. Another correlation was found between severity of depression and willingness to use an online platform, with a more pronounced depression going along with an increased willingness to use an online platform (*r*=.340, *P*=.01). There was a weak correlation between the degree of extraversion and the readiness for use (*r*=.272, *P*=.04). More extraverted people were more open toward using an online platform. Finally, another weak correlation could be found between the level of desired social support and the willingness to use an online care platform (*r*=−.226, *P*=.04); a higher level of desired social support was a sign of a higher willingness to use an online supply platform.

### Interviews

The text was pooled into the following 3 categories: resources, experience with online interventions, and prerequisites for a collaborative online care platform. First, it was assessed what helped interviewees in dealing with their depression. Most notable were social support, psychoeducation and hobbies, such as reading, sports, or volunteering, *but that only helps to a certain degree.* Another goal of the individual interviews was to examine what experiences with online therapy elderly patients with depression had. Although Person B reported having never used any form of online therapy, Person A had limited experience with online therapy. However, so far person A used the internet primarily for information and research purposes.

In addition, the question, “which criteria respondents consider a necessity for an online care platform to be effective?” was asked. Interestingly, Person A thought that some form of acute online instructions “such as lie down and listen to a bit of relaxing music, and then the person can listen to some for free” would be ideal. The following specific wishes were expressed: unlimited access, easy usability, psychoeducational information, opportunity to exchange information, motivation, platform-independent access, suitable for slow internet connections, and privacy.

## Discussion

### Principal Findings

Focus groups, online surveys, and interviews were conducted to allow an accurate assessment of the prerequisites and willingness to use a collaborative online care platform addressing the special circumstances and needs of the elderly, as well as the needs and requirements of those caring for them and treating them.

The results of the focus groups highlighted that there is a need for a collaborative care platform for depression in old age. However, it was already apparent in the focus group discussion that depressed seniors could be overwhelmed with computer-aided measures. Only future generations could have sufficient skills in their old age to make meaningful use of such offers. This picture was confirmed in the online survey. A comparison of the 2 age groups revealed that younger participants used electronic media more often than older participants. The findings are in line with previous research that has also shown different usage patterns between younger and older participants [35]. Nonetheless, the results of this study underline that the use of ICT was relatively widespread. Consequently, it can be concluded that online interventions could well have the potential [36].

A similar trend can be seen when analyzing the willingness to use: younger participants were more willing to use an online care platform. This finding can in part be explained by another finding of this study; more severe depression was associated with higher willingness to use online services. As younger patients showed more symptoms of depression, they might be more open to online therapy options. Nonetheless, this difference was also found in previous research on the role of age in the willingness to use online interventions [35]. It remains unclear whether in future, older patients will be more receptive because of greater ICT knowledge and experience, or whether they will show less willingness because of other barriers.

The positive correlation between willingness to use an online intervention and extraversion is no surprise, considering extraversion and the influence of this personality dimension on help-seeking behavior. Previous studies already established that extraversion is a predictor of help-seeking behavior in different life situations [36]. In addition, the positive correlation between the desired level of support and the willingness to use an online care platform reflects past research, indicating a link between social isolation and increased readiness to receive benefits [37].

Finally, the interpretation of the interviews points in a comparable direction. Although the younger participant not only had limited experience with online services, the older respondent had no idea of the possibilities of online therapy. From these very uniform findings, it can be concluded that older depressive participants (65 years and older) not only have very limited knowledge about the possibilities of an internet supply platform but are currently unwilling to use a corresponding offer.

With regard to prerequisites to use an online care platform, the focus group formulates particularly low costs, integration into everyday life, and easy handling as important features. In the user survey, privacy and data protection emerged as the most significant factors. Other features mentioned not only in the online survey but also during interviews included ease of use and free access. In addition, the opportunity to network with other stakeholders, as well as comprehensive information and support in acute crisis situations were highlighted. To conclude, the following factors can be summarized as particularly important for an online care platform: simple use, data protection, and costs.

### Methodological Limitations

There are some methodological limitations to this study. Due to the small sample size, the results of this study are to be interpreted with care. Only a few participants could be recruited both for the online survey as well as for the interviews. However, the reduced participation in epidemiological studies has been extensively shown in the literature [38]. In particular, response rates for telephone interviews have been decreasing rapidly [39]. This might be explained by an oversaturation of online forums and self-help groups with inquiries to take part in yet another online study. A more optimistic perspective might be that only a few visitors of investigated forums and websites belong to the target group. Therefore, the small number of responders represents the digital-savvy portion of this particular age cohort. As the usage of the internet is rapidly increasing, this study could be well understood as a road sign for a growing group in the future. These difficulties in recruiting subjects also confirm discussed results, in the sense that older depressive people are overwhelmed with online offers. Nonetheless, this study presents a comprehensive picture of the conditions and willingness to use a potential online care platform for elderly patients with depression. Further research on the willingness of older people to use e-mental health apps is necessary. For example, the willingness to use face-to-face therapy versus the willingness to use online services should be compared. It would also be important to measure the willingness to use eHealth services after participants were able to collect some experience themselves so that assessment can be based on concrete experiences and not on an abstract concept as was the case in this study.

### Conclusions

In conclusion, elderly patients with depression are willing to use a collaborative online care platform for depression. Age plays a significant role whereby younger elders are significantly more willing. When designing an online care platform, a particular focus should be on making the intervention simple to use, ensuring online privacy, and making the service as affordable as possible. Online care platforms seem to constitute a viable intervention for older patients. Further research is needed to extend our findings to the overall population.

## Abbreviations

BFI-10: 10-item Big Five Inventory
CBT: cognitive behavioral therapy
cCBT: computerized cognitive behavioral therapy
DSM: Diagnostic and Statistical Manual of Mental Disorders
e-mental health: electronic mental health
ICD: international classification of diseases
ICT: information and communication technology
PHQ: Patient Health Questionnaire
